# 'It's not just about fish': Assessing the social impacts of marine protected areas on the wellbeing of coastal communities in New South Wales

**DOI:** 10.1371/journal.pone.0244605

**Published:** 2020-12-30

**Authors:** Natalie Gollan, Kate Barclay

**Affiliations:** 1 Faculty of Arts and Social Science, University of Technology, Sydney, Broadway, NSW, Australia; 2 New South Wales Department of Primary Industries (Fisheries), Port Stephens Fisheries Institute, Nelson Bay, NSW, Australia; University of Bucharest, ROMANIA

## Abstract

Managing Marine Protected Areas (MPAs) is about managing human behaviours, but decision-making processes have traditionally focussed on ecological aspects, treating social aspects as secondary. It is now becoming more evident that an equal focus on the ecological and social aspects is required. Without the collection of information about social aspect such as impacts and sharing this as well as ecological information with communities, MPAs are at higher risk of opposition and social acceptability problems. This paper explores the development of a wellbeing framework to understand the social aspects, including the impacts of MPAs on the wellbeing of local communities. This research investigates two case study MPAs: Cape Byron and Port Stephens-Great Lakes Marine Parks in New South Wales, Australia. The MPAs are multiple-use and were implemented in 2006 and 2007, respectively. The research began with a review of the literature, followed by fieldwork, including semi-structured qualitative interviews with community members. Through thematic coding of the interview transcripts in light of the literature on assessing the social impacts of MPAs, a community wellbeing framework of domains and associated attributes was developed to investigate social impacts. Our analysis shows; first, local perspectives are crucial to understanding social impacts. Second, understanding social impacts gives insight into the nature of trade-offs that occur in decision-making regarding MPAs. Third, the intangible social impacts experienced by local communities are just as significant as the tangible ones for understanding how MPAs operate. Fourth, governance impacts have been the most influential factor affecting the social acceptability of the case study parks. We argue that failure to address negative social impacts can undermine the legitimacy of MPAs. We propose that the framework will support policymakers to work towards more effective, equitable and socially sustainable MPAs by employing much-needed monitoring of human dimensions of conservation interventions at the community level to shape adaptive management.

## 1. Introduction

Environmental degradation and loss of biodiversity globally have led to the establishment of conservation initiatives. These efforts resulted in the push for a more efficient integrated approach to marine ecosystem management, one component of which includes Marine Protected Areas (MPAs), many of which have been established globally to protect biodiversity [1]. An effort to meet the global Aichi Target 11 means that the numbers of MPAs are increasing over time [2], with a commitment by 2020 as part of the global Aichi Target 11 for at least 10 per cent of coastal and marine areas to be protected [3]. The Aichi target includes biodiversity but also the provision of ecosystem services (such as erosion control, protection of sacred sites) [4].

The most commonly accepted definition of a protected area, developed by the International Union for Conservation of Nature (IUCN), refers to ‘[a] clearly defined geographical space, recognised, dedicated and managed, through legal or other effective means, to achieve the long-term conservation of nature with associated ecosystem services and cultural values’ [5]. MPAs are an umbrella term for protected areas such as marine parks, aquatic reserves and marine reserves.

One of the conditions to meet the Aichi Targets is for MPAs to be equitably and effectively managed with participation from local communities and Indigenous peoples so that the distribution of costs and benefits are shared relatively [4]. Negative impacts (or costs) can include changes to culture, way of life and sense of place, while positive impacts (or benefits) can include enhancing food security [6–8].

Several reviews of social impacts of protected areas have been undertaken and highlighted that the vast majority of studies on social impacts have been narrow in focus, primarily dealing with objective measures, e.g. income or increase employment in the area [9–11]. In recent years, there has been an increasing amount of literature on social impact assessments, including subjective measurements, by asking local communities how they perceive the impacts. Subjective measurement is a necessary and critical component of social assessments, rather than using only conventional objective measures [9, 12–14], as it allows people to express how they feel about impacts. It raises impacts that may not be considered if only objective measures are used [15].

Despite the increase in studies, however, there is still an insufficient evidence base about the social impacts of MPAs [16–19]. The focus of most social impact research has been on extractive users directly impacted by MPAs, including commercial, Indigenous and artisanal fishers. There are still very few studies on other stakeholders, including non-fishing stakeholders, and local communities [8, 10].

Human wellbeing is a useful lens through which to view social impacts. In the last decade, there has been an increasing policy focus that conservation initiatives should improve human wellbeing, with many large conservation agencies shifting their vision statement to include a reference to people and wellbeing [20, 21].

Wellbeing includes the concepts of quality of life, life satisfaction, physical, mental and emotional health [22, 23]. Although there are many definitions of ‘wellbeing’, most agree that it requires meeting human needs, and the ability to pursue one’s goals and life satisfaction [24–26].

Two important theories around individual and community wellbeing include the capabilities approach, and the basic needs approach [27, 28]. Amartya Sen’s capabilities approach, developed in the 1980s, moves away from income to focus on substantive freedoms and quality of life [29, 30]. The capabilities approach recognises the importance of a person’s autonomy in achieving various ‘functionings’, i.e. ‘the various things a person may value doing or being’ [29 p. 75]. The basic needs approach includes, for example, Doyal and Gough’s Theory of Human Needs [31]. The theory of human needs identifies autonomy and health as universal elements for wellbeing. The theory differentiates between ‘wants’, which reflect the cultural environment, and ‘needs’, which are understood to be in theory applicable to all people [32].

A contemporary multi-dimensional wellbeing approach builds on the established theory. It brings together human needs, freedoms and quality of life that can include a diverse list of universal dimensions. The subjective dimension is an important aspect of the wellbeing framework for providing insights into people’s values, aspirations and feelings that allow for a more valid and accurate assessment of community wellbeing [19, 28, 33]. A wellbeing approach also gives an understanding and consideration of the different trade-offs between social, economic and environmental outcomes in decision-making that occur when implementing conservation interventions such as MPAs [34]. For example, trade-offs between the wellbeing outcomes of different user groups such as scuba/snorkel tourism operators benefiting from no-take zones but these zones can displace fishers impacting on their livelihood [35].

Wellbeing studies in natural resource management have been used in risk assessments for understanding threats to community benefits [36]; assessing the wellbeing of fishing communities [25]; measuring human wellbeing as part of social-ecological systems [37, 38]; and assessing ecosystem services including the non-material benefits (e.g. spiritual, aesthetic) that people gain from the environment [39].

A recent synthesis of the impact of MPAs on human wellbeing [11] found that the consideration of the different domains of wellbeing was uneven with the economic, governance and environment domains the most commonly studied, and social, health and cultural domains the least studied. They analysed 118 papers globally and found more positive wellbeing outcomes (51%) than negative wellbeing outcomes (31%) across stakeholder groups. Recreational users were understudied. Rasheed (2020) undertook a systematic review of MPAs and wellbeing and found research on human wellbeing in MPAs is limited, with empirical studies rare [10].

**Fig 1:**
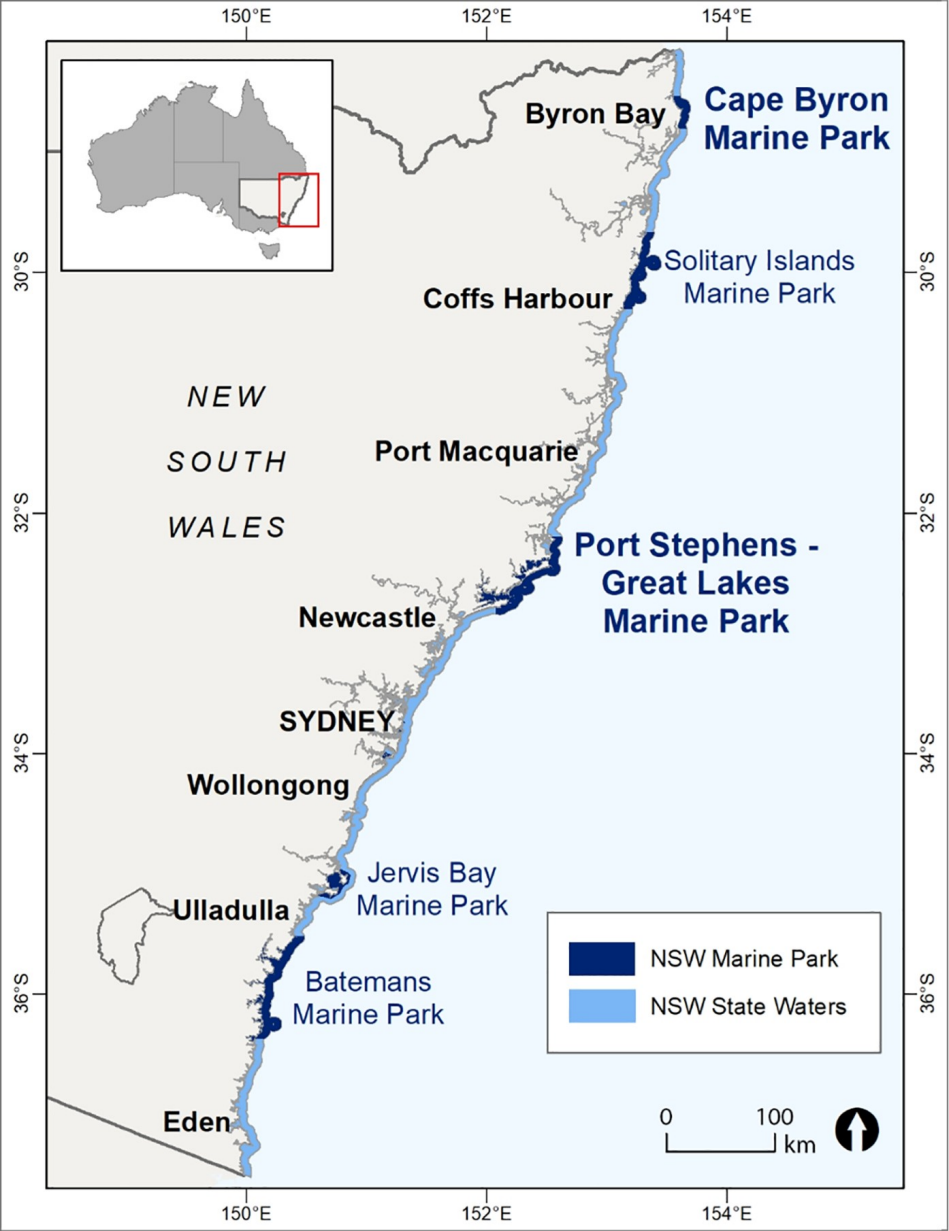
Map of NSW marine park including the case studies: Cape Byron Marine Park and Port Stephens-Great Lakes Marine Park. Reprinted from DPI under a CC BY license, with permission from DPI, original copyright 2020.

This paper presents empirical wellbeing data about two marine protected areas from local communities adjacent to Cape Byron Marine Park (CBMP) and Port-Stephens Great Lakes Marine Park (PSGLMP) in NSW, Australia (Fig 1). This research is one of the first empirical applications of a wellbeing framework in a local MPA context considering social impacts experienced by a range of interest/user groups, not just extractive users. In this paper, the differences in wellbeing between different interest/user groups are not explored. Instead, the aim is to give an overall view of the wellbeing framework, methods and the types of insights it can reveal on the range of social impacts MPAs can have on local communities.

The paper first sets out the wellbeing framework used in this research, then moves on to present interview data about the social impacts concerning each domain of wellbeing: environment; health and safety; social connections; education and knowledge; culture and heritage; governance; and local economy. The paper concludes with insights on developing much-needed monitoring of human dimensions of conservation interventions at the community level.

## 2. Material and methods

The research project received full ethics clearance from the University of Technology Human Research Ethics Committee on 5 April 2017, approval number UTS HREC ETH16-1080.

### 2.1 Conceptual framework

A multi-dimensional wellbeing approach overcomes some of the shortcomings and challenges of other approaches. In particular, it examines all aspects of wellbeing, captures the diversity of stakeholders and assesses intangible impacts, e.g. values, aspirations, and cultural traditions.

An iterative approach using a combination of top-down (from the research literature) and bottom-up (from local primary data) approach was chosen as the most suitable methodology for this research as it means the framework is grounded in local contexts but also builds on existing theory and can be applied in other contexts.

The domains of wellbeing were derived from the literature review and primary research in the form of semi-structured interviews. A qualitative approach to this study was employed drawing from participatory wellbeing methodology [40–42] whereby individuals feel empowered by having their voices heard. Participatory methods are important where resources are used by diverse users with competing interest and values [43, 44]. The domains were unpacked by further disaggregating them into attributes. Impacts were categorised in the relevant attribute/domain.

In some cases, the attributes are inherently interrelated with other attributes. For example, food can belong in a domain about health as well as a domain relating to culture and heritage. Attributes, however, were placed in just one domain to reduce double-counting for monitoring of the impacts. Social impacts were derived from the primary data, as impacts should be defined by the communities who have been impacted [45]. A broad framing of social impacts was adopted, meaning issues such as governance processes are included as impacts.

Social impacts in adjacent communities from the establishment and ongoing management of CBMP and PSGLMP was previously not known. Categorising impacts within a wellbeing framework is useful as it captures the complexity of wellbeing as well as tangible and intangible aspects.

### 2.2 Case study sites

There are several factors to be considered when selecting case studies, including practical considerations such as data availability, good sources of documentary evidence, as well as relevance and usefulness [46]. The two case studies explored in-depth the social impacts of two marine parks that are bounded by time (data collection of 18 months) and place (Cape Byron Marine Park and Port Stephens-Great Lakes Marine Park) [46, 47].

Cape Byron Marine Park is located on the NSW far north coast from Brunswick River to Lennox Head. Port Stephens-Great Lakes Marine Park is located in central NSW, extending from Cape Hawke near Forster south to Birubi Beach at the northern end of Stockton Beach (Fig 1). The implementation of the zones and management rules for the parks were gazetted within two consecutive years, 2006 for Cape Byron and 2007 for Port Stephens-Great Lakes marine parks and at the time of selection the marine park management plans were not going through a Government review. We did not want to choose parks that were subject to government review during the research period as it would introduce sensitivities that could make the research difficult.

Both are multiple use marine parks, with a range of zones from sanctuary–providing for the highest level of protection–to general use–providing for a wide range of uses (Table 1).

**Table 1:** Zones in Cape Byron and Port Stephens-Great Lakes Marine Parks and summary of uses permitted (source: Collaborative Australian Protected Areas Database (CAPAD) 2018, commonwealth of Australia 2019).

Zone types	Summary of uses	CBMP (% of total area)	PSGLMP (% of total area)
Sanctuary (IUCN II)	Provides the highest level of protection by only allowing activities that do not harm plants, animals or habitats	27.5%	17.9%
Habitat protection (IUCN IV)	Help to conserve marine biodiversity by protecting habitat and reducing high impact activities	19.2%	38.2%
General use (IUCN VI)	Provide for a wide range of environmentally sustainable uses	53.2%	43.8%
Special purpose IUCN VI)	Provides for specific management arrangements including Aboriginal culture, marine facilities or for specific park management reasons	0.1%	0.1%

### 2.3 Data collection and analysis

The research began with a pilot study to test the preliminary theoretical framework. The pilot study consisted of face-to-face semi-structured interviews—with seven participants in each marine park selected from the parks’ advisory committee. The interviews took place in each marine park. Participants were asked in an open-ended manner to say the positive and negative impacts of the MPAs on their wellbeing and that of local communities living in areas adjacent to CBMP and PSGLMP. The pilot study enabled an initial understanding of wellbeing in coastal communities in NSW. After initial analysis, the interview questions were restructured to allow for a greater exploration of the nature of people’s experiences of the impacts of MPAs. Interviews were between thirty to ninety minutes in length, audio-recorded and then transcribed verbatim at the earliest opportunity [48].

The research uses a multi-interest/user group approach to ensure that people with a wide range of interests in MPAs from adjacent coastal communities were interviewed. The participants were selected by ‘purposive sampling’. In the first instance, for the pilot studies, participants interviewed were selected from the CBMP and PSGLMP advisory committees. The government chooses these advisory committees to allow all segments of local communities to participate in the management of their local marine park. In addition to interviewing advisory committee members, additional participants were recruited by the ‘snowball’ sampling technique [49]. The overall patterns of use across all participants were considered in making sure each type of use was represented by several interviewees (Table 2). It is important to note that many interviewees interact with the coastal-marine environment in more than one way; for example, a commercial fisher participant also snorkelled, spearfished, surfed and collaborated in the research. In total, 58 people were interviewed from the two parks, spanning interest groups across extractive and non-extractive, active, passive, commercial and community uses.

**Table 2:** Overview of interest/user groups in Cape Byron and Port Stephens-Great Lakes Marine Parks (adapted from [51]).

	Active uses	Passive uses	Commercial uses	Community uses
Extractive use	Recreational fishing [18]	Rock pool or platform foraging [2]	Professional fishing/aquaculture [9]	Sport competitions (e.g. fishing competitions) [2]
	Spearfishing [2])			
	Power boating, water skiing, jet skiing [3]		Charter fishing [2]	
	Subsistence and Aboriginal fishing [7]			
Non-extractive use	Swimming, surfing, boarding [17]	Walking, exercising, sunbathing [11]	Commercial dive tours [3]	Undertake educational activities or scientific research [13]
	Kayaking/canoeing [6]	Wildlife appreciation activities [15]	Business: tourism and retail industries [18]	Voluntary environmental work [5]
	Scuba diving/snorkelling [19]	Sailing [3]	Whale and dolphin watching charters [3]	Aboriginal ceremonial and cultural use [7]
		Pet exercising [5]		Sport competitions (e.g. surfing) [1]

*Note: The numbers in brackets are interviewee numbers for each group. Participants were able to nominate more than one use, so the numbers in this table exceed 58.

After transcription, multiple rounds of coding were conducted by a single coder (Gollan) systematically reading through the transcripts. NVivo qualitative analysis software was used to code transcripts and notes. The first cycle of coding across the whole dataset generated initial codes for domains, attributes and impacts. The second cycle of coding was pattern coding which identified potential themes and grouped the codes into a smaller number of themes [48]. The next step was thematic analysis to identify themes and patterns of meaning across the data. Thematic analysis is flexible and provides a rich analysis of qualitative data, such as interviews [50]. A codebook listing themes and descriptions were generated and used to provide consistency across the data set.

## 3. Results

### 3.1 Community wellbeing framework

Wellbeing is multi-dimensional, and in the context of this study, interviews indicated that the marine and coastal environment contributes to many domains of wellbeing. These include health (such as mental and physical health benefits of surfing, fishing); social connections (such as spending time with family and friends in nature); education and knowledge (teaching children to appreciate nature through activities such as snorkelling); culture and heritage (such as Aboriginal people’s access to Country, freedom to practice culture and pass down knowledge to younger generations); governance (such as participation in decision making); and local economy (such as providing livelihoods). A healthy marine and coastal environment underpinned the wellbeing of individuals and communities adjacent to CBMP and PSGLMP.

**Fig 2:**
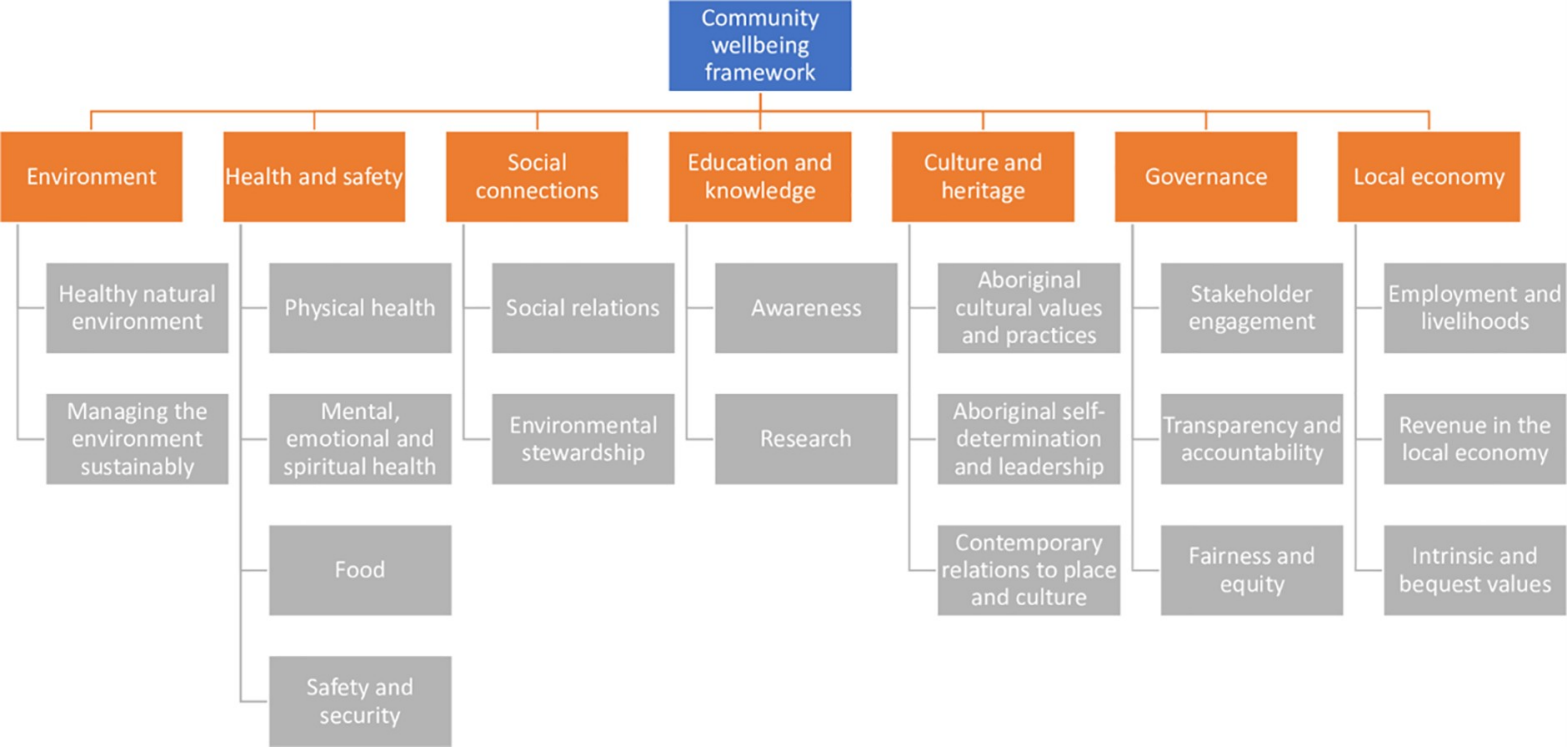
Community wellbeing conceptual framework.

Seven domains of wellbeing were identified from participant interviews and the literature [37, 38, 52–54]: 1) environment; 2) health and safety; 3) social connections; 4) education and knowledge; 5) culture and heritage; 6) governance and 7) local economy (Table 3, Fig 2). Local communities living adjacent to CBMP and PSGLMP experienced a wide range of positive and negative impacts associated with the establishment and ongoing management of the MPAs. The framework helps us understand the social impacts of MPAs, consisting of seven domains of wellbeing, 19 attributes, 25 positive impacts and 29 negative impacts.

**Table 3:** Domains of wellbeing relevant to marine protected areas.

Domains of wellbeing	Description
Environment	The biophysical environment is the source of our continued wellbeing and is essential to the quality of life of individuals and broader society. The marine and coastal environment contribute to our wellbeing in many ways, including beauty, clean water and food production [12]. For Aboriginal people, the environment ‘Country’ is part of who they are; it gives them their cosmology, identity, stories and shapes their languages [55, 56].
Health and safety	The health benefits associated with contact with nature, such as fishing, passive activities, and nature-based education can benefit human health [57, 58]. Community members can perceive MPAs as a safe space to spend time with family and friends [59]. Conversely, MPAs can impact negatively on safety by closing local fishing grounds, meaning fishers must drive further to access fishing spots.
Social connections	Social connections include the social relationships strengthened through activities conducted in the marine and coastal environment, including between family and friends, other groups of people (for example, work colleagues) and government agencies. The parks can increase socialising with like-minded people by creating safe snorkelling spots but also can decrease socialising through, for example, reduction in fishing club membership due to the closure of local fishing grounds.
Education and knowledge	Education can improve awareness and understanding of the natural environment and can influence people’s attitudes and behaviours towards natural resource management [60, 61]. Research benefits of MPAs include the gathering of knowledge from no-take zones, providing rich sites for nature-based education [61] and contribute to evidence-based research.
Culture and heritage	Aboriginal and non-Aboriginal attributes of culture and heritage include cultural values and practices related to the environment and sense of place. The implementation of MPAs can impact on culture and heritage by preserving areas for activities allowed within the parks, but also by restricting access to areas or activities that are culturally important.
Governance	MPAs are spatially defined governance systems that have their own sets of rules and regulations. Governance is an essential domain of wellbeing for communities because participation, trust, fairness and equity in marine and coastal management processes can impact on people’s quality of life [11, 62].
Local economy	The economic domain is one of the more familiar domains for measuring human wellbeing. In many coastal areas, the local economy is closely tied to the health of the marine and coastal environment. The marine environment supports industries including fishing, aquaculture, marine tourism and recreation activities, offshore oil and gas exploration and extraction, boat/shipbuilding, repair and maintenance and infrastructure [63]. These industries can be impacted positively and negatively by MPAs.

### 3.2 Environment domain

The environment is essential to consider when understanding social impacts, as although it is mostly assessed in ecological terms, people also place social and cultural values on the environment. In other words, loss of habitat or threatened species is a social impact as well as an ecological one [45]. Based on the results of coding semi-structured interview data, the environmental domain was disaggregated into two attributes: 1) healthy natural environment; and 2) managing the environment sustainably. Five key positive impacts and two negative impacts (Table 4) emerged from the analysis across both MPAs.

**Table 4:** Environment domain and associated attributes and corresponding social impacts (positive and negative) of Cape Byron and Port Stephens-Great Lakes Marine Parks.

Environment dimension			
Attribute	Impacts (positive or negative)	Number of coding references (i.e. number of ‘mentions’ in interviews)	Number of interviews coded at this theme (n = 58)
**Healthy natural environment**	Biodiversity protected	80	34
	Threatened and protected species protected	13	10
	Natural beauty and value protected	41	28
	Enjoy observing marine life at no-take zones	28	15
**Managing the environment sustainably**	Local threats to the marine environment reduced, e.g. by regulating extractive users	57	29
	Local threats not reduced, e.g. allowing extractive users	22	10
	Increased fishing pressure on areas adjacent to no-take zones	7	4

The recognition of a healthy environment underpinning the wellbeing of participants came up in discussions of why participants lived near the marine environment. Over half of the interviewees perceived the marine park as contributing to the coastal community through, for example, protecting biodiversity and threatened and protected species. As one participant said:

It's an environment that is, year on year you get the leopard shark return and manta rays return during the summer months. The grey nurse shark returns during the winter months. We've always got turtles out there. We've got hundreds of fish out there. So, it's great to see that as a scuba diver to see such incredible habitats that are really that healthy. (CBMP_8)

A recurrent theme in the interviews was a sense amongst participants that the health of the environment was degrading globally, and MPAs are a management tool that reduces local threats to the marine environment. For example, one participant said:

And even though it's only 4% of the coastline, I mean, at least that's- imagine if we did a little more. Imagine the impact then. And from a diving point of view, it's awesome to go down into a sanctuary where there's no fishing line, fish hooked in rocks and cleaning up rubbish and bottles. So that's kind of the fantastic part about the marine park themselves. Worth protecting. (PSGLMP_19)

About one-quarter of the participants, mainly recreational users value observing marine life in no-take zones (unfished sites), seen as a positive impact that makes the experience of the marine park through the enjoyment of viewing or being surrounded by marine life, as one individual stated:

So now when you see a big bait ball cruise past and you see dolphins, gannets, everything having a feed, and you’re like, how nice is that that they get first pickings, second pickings, and that they don’t have to compete with the fishos, with humans anymore. Yeah, it’s a nice feeling. (CBMP_ 12)

MPAs are valued by participants predominantly for their environmental protection, providing habitat for marine life, conserving threatened and protected species. When asked if they think the marine park has been effective in managing those threats, many understood that the park cannot stop, for example, urban runoff or climate change, but could stop threats such as netting of fish. Many believed that the park could address some local threats to build resilience.

In contrast, some participants, primarily extractive users, perceived that the marine parks cause environmental damage through increased fishing pressure in adjacent areas. As one participant put it:

Unfortunately, the marine park has stopped me accessing areas in a southerly, accessing areas in a south wester, and it meant that my effort has shifted, and I consolidated my effort in smaller areas. And so, I think the marine park effectively made people’s effort consolidate and its created overfishing in areas that were previously not overfished. (PSGLMP_22)

Consequently, they believe that a management regime other than marine parks is required, e.g. changes to bag and size limits, and reducing destructive fishing methods.

### 3.3 Health and safety domain

The health and safety domain was disaggregated into four attributes; 1) physical health; 2) mental, emotional and spiritual health; 3) food; and 4) safety and security. Three key positive impacts and six negative impacts emerged from the analysis across both MPAs (Table 5).

**Table 5:** Health and safety domain and associated attributes and corresponding social impacts (positive and negative) of Cape Byron and Port Stephens-Great Lakes Marine Parks.

Health and safety domain			
Attribute	Impacts (positive or negative)	Number of coding references	Number of interviews coded at this theme (n = 58)
**Physical health**	Increases physical activity related to MPA (e.g. snorkelling, surfing)	21	16
**Mental, emotional and spiritual health**	Improves spiritual, emotional and mental health	18	12
	Increases mental health issues and stress	10	4
**Food**	Reduces locally sourced seafood from industry	18	10
	Reduces subsistence fishing	14	9
**Safety and security**	Increases safe spots to snorkel	3	2
	Reduces safe fishing spots	12	8
	Requires further travel to fish	10	5
	Antisocial behaviour and unsafe practices	23	11

The interviewees, overall, reported positive health benefits through connection to the marine and coastal environments within MPAs. Protected areas can contribute to physical and mental health by preserving the natural environment for its intrinsic value and inspiration. The comments below illustrate the importance of physical activity in nature for participants:

It's a healthy activity. Surfing sort of keeps you relatively fit and healthy. I think just the natural beauty of the place is just still sort of awe inspiring. You can be in parts of Byron Bay and see nothing but the natural environment in the water and the headland and hardly see a building. (CBMP_9)

For about one-quarter of the participants, the MPAs contributed to their emotional and spiritual health, as one interviewee put it:

It's just that spiritual side, to start with. You get out on the water, in the water, you feel good [laughter]. You just feel good. You see a bunch of—200 common dolphins, or 10 whales, like we did today, you just feel good. It feels good. It's better than sitting in an office somewhere. (PSGLMP_21)

However, in some instances, the implementation of MPAs by restricting people’s access can have adverse effects on emotional and spiritual health. Some participants felt that the implementation of the parks disregarded historical ties to the area and the impact the MPAs have had on the emotional health of some stakeholders, mainly commercial fishers. As one participant said:

Historical fishing families have experienced loss of work and income after five generations of managing the lake… total disregard of the emotional impact and historical ties to the profession. (PSGLMP_7)

Cultural fishing is integral to the lives of Arakwal (CBMP) and Worimi (PSGLMP) Aboriginal peoples. Cultural fishing is spiritually important and has contributed an essential part of the diet of Aboriginal people for thousands of years. As one interviewee put it:

Always been here, always lived here, yeah. My ancestors actually were the Worimi here in Port Stephens. So yeah, and we still live on our Country here and I guess our main activity would be fishing. So that was really important to the Worimi people. It was part of their dietary requirement, and the ancestors done that, and we carry that on. (PSGLMP_17)

MPAs restrict local fishing grounds which can have a negative impact on local communities’ access to seafood. For example, one participant described the impact that the PSGLMP had on the diet of some Aboriginal people of Port Stephens:

Dad used to eat mullet when he was young and even when he had kids when I was young, he used to eat mullet five times a week. So, he used to go and net the rivers and feed the family that way five times a week, five meals. Now he is lucky to eat it five times a year because netting needs a permit and it takes a while to get a permit and then net and the license and the opportunities to do that we have now to do that in locations and different spots and all of sudden his diet had changed. And all the diet in the old people has changed. (PSGLMP_12)

In most cases, the interviewees reported that the MPAs contributed to a sense of safety in their community. As one participant said:

But I think just the sense that it feels like a safe place. The beaches are clean, the waters are unpolluted, I don't live in fear of having been broken into at home. It seems to be like a village, so people look after each other a bit more, care about it. There is a community spirit here. A lot of people are involved in voluntary organisations that take care of the place and each other. (PSGLMP_20)

However, some extractive users reported the parks negatively impacted their safety and security through the need to travel further distances to access fishing spots. Talking about this issue, one interviewee said:

When you are putting the boat in and out the beach in waves the bigger the boat the more hassle dangerous it is for you, you know. But that’s what they have forced us to do because you’ve got to go bigger distances you know. That’s just stuff that people don’t realise. (PSGLMP_6)

### 3.4 Social connections domain

The social connections domain is disaggregated into two attributes: 1) social relations and 2) environmental stewardship. Three positive impacts and one negative impact (Table 6) emerged from the analysis across both MPAs.

**Table 6:** Social connections domain and associated attributes and corresponding social impacts (positive and negative) of Cape Byron and Port Stephens-Great Lakes Marine Parks.

Social connections domain			
Attribute	Impacts (positive or negative)	Number of coding references	Number of interviews coded at this theme (n = 58)
**Social relations**	Facilitates community cohesion	24	16
	Increases socialising with like-minded people	11	6
	Reduces socialising with like-minded people	7	2
**Environmental stewardship**	Increases positive attitudes towards the environment and behaviours to protect it	38	20

Most participants across the different interest/user groups indicated that the marine and coastal environment within CBMP and PSGLMP had a positive impact on social relationships with friends and family and community. Just over one-quarter of the participants said that MPAs create community cohesion through people collaborating in caring for the place, with marine parks becoming ‘part and parcel’ of the whole community.

The management of MPAs can enhance the collaboration and active involvement of people in measures to work together to protect the marine environment. Environmental stewardship is about individuals and communities getting involved to promote sustainability and is evident in both parks, such as creating a Marine Parks Association that is specifically engaged to support marine parks.

Increasing socialising with like-minded people was raised by recreational users, with MPAs creating spaces where, for example, snorkelers and divers can gather and enjoy the activity without conflict with fishers fishing over them.

However, many of the recreational and commercial fishers are vehemently opposed to any intervention that restricts their fishing. Loss or restricted access is not just about the loss of physical access to fishing spots, but also has a range of unintended consequences, including the loss of socialising with like-minded people which was felt by a small number of interviewees. For example, the local fishing club at Seal Rocks was seen to have significantly reduced in size as a result of the implementation of PSGLMP. The reduction of fishing club members was perceived as explicitly due to the loss of access due to the sizeable no-take zone (the largest no-take zone in the PSGLMP). As one interviewee put it:

We went from a club with about 15 boats, fishing once a month down to three, because of the marine park they just won’t fish it. (PSGLMP_6)

### 3.5 Education and knowledge domain

The education and knowledge domain were disaggregated into two attributes: 1) awareness; and 2) research. Four key positive impacts and two negative impacts emerged from the analysis across both MPAs (Table 7).

**Table 7:** Education and knowledge domain and associated attributes and corresponding social impacts (positive and negative) of Cape Byron and Port Stephens-Great Lakes Marine Parks.

Education and knowledge domain			
Attribute	Impacts (positive or negative)	Number of coding references	Number of interviews coded at this theme (n = 58)
**Awareness**	Increases awareness of the marine and coastal environment (including benefits/threats to the marine park)	26	15
	Lack of education and awareness of the benefits/threats of the marine park	63	30
	Lack of education and awareness of the significance of Aboriginal Sea Country values	14	8
	Children’s enjoyment creating opportunities for education	8	6
	Increased education opportunities and awareness of local ecological knowledge	17	11
**Research**	Provides baseline data on unfished systems	12	8

Education was a recurrent theme in the interviews, and there was a sense amongst interviewees that education is key to the success of the marine park and community ‘buy-in’. Participants perceived that a marine park is a tool for creating environmental awareness; it allows fisheries enforcement to be ‘front of mind’ when people are doing their extractive activities. About one-quarter of the participants perceived that local communities are more educated about the marine and coastal environment because of the parks. As one interviewee said:

I think the best thing about the marine park is community awareness that we’ve got to look after what we’ve got. You know, that is something special and I think now the community appreciate that there is a marine park. I know it was controversial when it was introduced, but I think if you take it away now from people there would be protest because people have learned to appreciate it. (PSGLMP_5)

Over half of the participants perceived that there was a lack of education on the environmental benefits of the parks inhibiting the positive impacts. As a result, some participants feel that the community are ignorant of the marine park and do not care.

Research can contribute to community wellbeing through learning and promoting benefits of the marine environment, with some participants valuing that baseline data is being collected on unfished ecosystems. As one participant put it:

If research is being undertaken, and it's providing benefits, and the community are becoming aware of it, I mean, that's a positive thing I think as well for the community to know that research is happening in our marine park by specialists in certain areas and they're finding out information and that's contributing to moving forward with marine park. I think that's a really important thing. (PSGLMP_11)

### 3.6 Culture and heritage domain

The culture and heritage domain were disaggregated into three attributes: 1) Aboriginal cultural values and practices; 2) Aboriginal self-determination & leadership; 3) contemporary relations to place and culture (Aboriginal and non-Aboriginal). Six key positive impacts and three negative impacts emerged from the analysis across both MPAs (Table 8).

**Table 8:** Culture and heritage domain and associated attributes and corresponding social impacts (positive and negative) of Cape Byron and Port Stephens-Great Lakes Marine Parks.

Culture and heritage dimension			
Attribute	Impacts (positive or negative)	Number of coding references	Number of interviews coded at this theme (n = 58)
**Aboriginal cultural values and practices**	Protection of cultural values and practices	16	7
	Loss of values and practices	16	7
	Loss of access to culturally significant places/marine resources	20	3
**Aboriginal self-determination and leadership**	Increases participation in Sea Country management	13	4
	Lack of self-determination and leadership in management	7	5
**Contemporary relations to place and culture (Aboriginal and non-Aboriginal)**	Enhances connection to nature	30	22
	Place attachment associated with the marine park	63	33
	Identity associated with the marine park	42	25
	Pride in the marine park	30	23

Participants conveyed a variety of perspectives relating to Aboriginal cultural values and practices including spiritual beliefs, customs, lore, languages, art and responsibilities. Aboriginal people have a cultural responsibility to protect and preserve Sea Country. Aboriginal and non-Aboriginal participants perceive that the marine park is protecting Aboriginal cultural values such as cultural fishing. As one participant commented:

I think it’s needed [the marine park] in Port Stephens because it’s a tourist destination. And if we can protect as much as we can, not just for the generation now, but for the future. So, my people can still go out and go to their cultural fishing areas and still get a feed of fish for the day. (PSGLMP_17)

However, a standard view amongst Aboriginal participants interviewed was the loss of access to traditional areas resulting in loss of connection to Sea Country. Cultural beliefs and knowledge such as storytelling can impact on the wellbeing of Aboriginal people, inhibiting the living culture. As one Aboriginal participant commented:

So, it’s that type of passing on the information in different areas, and teaching the youth, and keeping this continuation of our stories and our storylines and our culture–that’s kind of slowed down. I wouldn’t say gone, but all of a sudden, we can’t do anymore since the inception of it [the marine park]. So, it’s kind of prohibiting that continuation of our cultural practices and acts and stopping our storylines where we’ve got to sit back and talk about it instead of going there and living the culture with the kids. (PSGLMP_29)

Self-determination and leadership in decision-making are vital for the wellbeing of Aboriginal people. Aboriginal people aspire to culture-based governance models in CBMP and PSGLMP, including cultural considerations in policy development. Aboriginal participants in the parks feel disempowered by a top-down MPA governance system and want to self-regulate resource use. The comment below illustrates current MPA management as an inflexible approach to Aboriginal culture and Aboriginal peoples’ pursuit of self-determination. Presently, permits are required by Aboriginal people to fish in culturally relevant no-take zones, particularly in PSGLMP.

*I remember Dad saying for years that we couldn’t go to that spot*, *or particular spots*, *because a marine park was there where his father took him*. *And that happened for about 10 years*, *I remember*, *and we weren’t allowed to go into those areas*. *Since then we’ve had the access to permits*, *so we can go in there with a permit*. *That hasn’t been easy to get*. *It’s getting easier now*, *but it’s still asking a government organisation*, *White people*, *whether my father can teach his culture to his grandkids*. (PSGLMP_12)

CBMP and PSGLMP have special meanings for many of the non-Aboriginal participants. Activities in the parks contribute to a person’s sense of place and have positive or negative impacts on the wellbeing of participants, depending on the activities that are allowed. If the activities are those that are allowed in an MPA, the effects can be positive. Many of the participants described an emotional attachment to place, an increase in community pride with the MPA. Talking about this, one participant said:

A sense of pride, I guess, in living in an area where there's a marine park. It provides myself and the family with opportunities to do the activities that we want to do in our life. (PSGLMP_11)

### 3.7 Governance domain

Governance is an essential domain of wellbeing for communities because participation, trust, fairness and equity in MPA management processes can impact on a person’s quality of life. The governance domain includes the attributes: 1) stakeholder engagement; 2) transparency and accountability; 3) fairness and equity. One positive impact and 14 negative impacts emerged from the analysis across both MPAs (Table 9).

**Table 9:** Governance domain and associated attributes and corresponding social impacts (positive and negative) of Cape Byron and Port Stephens-Great Lakes Marine Parks.

Governance dimension			
Attribute	Impacts (positive or negative)	Number of coding references	Number of interviews coded at this theme (n = 58)
**Stakeholder engagement**	Inadequate engagement	48	22
	Ignored or disempowered	78	40
	Local ecological knowledge not valued in decision making	41	13
	Loss of trust	21	12
**Transparency and accountability**	Lack of scientific evidence	36	15
	Poor communication of science and management	66	28
	Unsatisfactory monitoring and evaluation of MPA effectiveness	80	33
	Questioning the legitimacy of MPAs	50	19
	Lack of confidence in management due to political interference	42	22
**Fairness and equity**	Creates community division through inequity of use	64	24
	Persecution, unfairly punished	22	10
	Impacts personal rights e.g. freedom	21	12
	Increases conflict between user groups	14	5
	Reduces conflict between user groups	36	18
	Loss of access to marine resources	41	21

Many (over two-thirds) of the participants felt ignored and disempowered throughout the ongoing management of the parks. Commercial and recreational fishers thought that the government had dismissed their concerns and left them with limited avenues for action. This perception came across similarly, if less strongly, from tourism operators, Aboriginal people and recreational users.

Participants said there are no avenues or channels for them to participate in decision making. Many stakeholders perceived that decisions are made externally, with no involvement of local people. The comment below illustrates participation decision problems, common in both parks.

For fishers, we just weren’t represented at all. In no way, shape or form. They were reinstating the same people [on the marine park advisory committee] over multiple terms. That was another massive insult. And then having no way to be able to–we’d send letters through to the chair, and then we’d just get brushed off. So, having no avenues or channels to be able to communicate. (CBMP_15)

There was a general belief from participants that the management of MPAs in NSW is not participatory, with decision making being top-down with little involvement from the public. As one participant put it:

The management of the marine estate in NSW falls right at the bottom just declare, advise and defend. I don’t believe there’s genuine engagement. (CBMP_3)

The lack of participation and deficient engagement leads to lack of trust between government and stakeholders. There was a sense of lack of confidence amongst some participants and the management of parks, as one participant commented:

So, it's highly conflicting interest there. And then fishermen get despondent, they lose a bit of confidence in the department; they lose a bit of confidence in the whole process. I don't know one fisherman that is opposed to a marine park in principle, but I know many who are opposed but because of the administration of it or how it has been administered. (PSGLMP_23)

Local ecological knowledge is an essential attribute of a person’s wellbeing as it allows for local communities to share their knowledge and needs and build local knowledge in MPA management processes. This attribute is inherently interconnected to the education and knowledge domain but has more relevance in this domain because it is about the use of knowledge in management. About one-third of the participants felt that their knowledge was not valued or incorporated into the MPA management processes, which had an impact on their wellbeing. Talking about this issue, one participant said:

It’s like we don’t exist which is sad because we see more stuff in that ocean because we’re out there. We’re picking up junk. We’re seeing how many whales are out there out wide. The numbers of fish that are there or aren’t there. But nobody cares or nobody even asks us. We’ve got no voice. We’re voiceless. (CBMP_19)

Stakeholders expressed a variety of perspectives in terms of monitoring and evaluation. There was a widespread perception by over half of the interviewees of unsatisfactory monitoring and evaluation programs in MPAs. This perception has led–primarily extractive users–to question the role of parks and if they should even be there at all. Moreover, many of the recreational users also perceived that there were unsatisfactory monitoring and evaluation of MPA effectiveness, inhibiting the positive impacts of MPAs. For example, one interviewee said:

I think the main thing that is lacking is more sort of research information about just how effective the sanctuary zones are. I think that sort of monitoring and assessment should be taking place in all of the sanctuary zones because like I say right now it’s just a gut feeling that things are ok, but I would like to see more actual monitoring to tell you whether it is or not. (PSGLMP_2)

Although there has been a strong emphasis on biophysical research conducted in both parks, the outputs are not easily accessible to the community, with scientific information primarily disseminated through scientific journals [e.g. 64–67]. The lack of communication of scientific outputs has contributed to the sense amongst some participants that there is no science behind the MPAs, as the comment below illustrates:

But it concerns me that everybody will go out and say these things are working, we’re doing a wonderful job, but there’s no evidence, there’s no proof. They’ve got no science behind it, it’s just a feel-good thing. (CBMP_7)

A possible explanation for the negative governance impacts is that alongside the implementation of the parks, the NSW Government committed to monitoring, evaluating and adaptive management through modifying zones. The government committed to a statutory review for the zoning plan for CBMP and PSGLMP for 2011 and 2012 consecutively to allow stakeholders and community to review the zoning plan after five years [68, 69]. It has now been well over a decade, and both parks are still yet to undergo a statutory review. From the government perspective, there is a reason for not having undertaken the promised reviews. A ‘new approach’ announced by the government in 2013, included the development of marine park management reforms involving changes to governance that replaced the existing NSW *Marine Parks Act 1997* with the *Marine Estate Management Act 2014* [70]. As a consequence of the new Act, the requirement of a statutory review of management rules for MPAs is now ten years since the enactment of that Act (i.e. 2024 for all NSW marine parks, not 2011 for CBMP and 2012 for PSGLMP as previously promised). Despite the government reforms, there is still an expectation in the community that the reviews pledged for 2011 and 2012 should have happened, which has led to social acceptability issues across different interests/user groups, as one interviewee commented:

Well the saddest thing about the marine park, well there’s lots of sad things, but the saddest thing is that we were promised a review after five years and no review. And it’s a long way from being perfect, a long way. (PSGLMP_3)

The perception of lack of confidence in management due to political interference was widespread across both parks, with some participants stating the decision-making based on politics was one of the greatest threats to the MPAs, as one participant commented:

I think the biggest threat to marine parks is political and the will of politicians to be influenced by people who have vested interest in getting things done, what they want particularly done. (CBMP_9)

Participants raised concerns about fairness and equity in PSGLMP relating to uneven distribution of negative impacts. For example, in PSGLMP, it was perceived that more significant negative impacts were experienced at the northern part of PSGLMP, as one fisher put it:

There's no two ways about it. They never got any massive closures at all in Nelson Bay. They got little, tiny pockets and we [Seal Rocks] copped the whole—we copped something like 85% of the sanctuary zones that were in our electorate. And they didn't cop anything down there, only little, tiny pieces….So there's a lot of discontent. It really, really wasn't done right. (PSGLMP_25)

Resource use conflict can be addressed in MPAs through the restrictions of certain activities, e.g. jet skis in CBMP, which was perceived as a benefit for many of the interviewees in CBMP. Alternatively, resource use conflict can be addressed through designating areas for specific users, e.g. snorkelers and divers in no-take zones, which was perceived as a benefit to recreational users. As one participant said:

The positive is especially down at the Moat at Lennox Head, which is a really popular snorkelling, and really safe snorkelling spot. There’s no fishing allowed there, and I think there’s great benefits in relation to people being able to snorkel, and kids being able to snorkel without the sort of conflict of having people fishing where you’re snorkelling. So, I think that’s a really positive outcome. (CBMP_14)

However, marine parks can also exacerbate conflict by restricting access to certain areas, e.g. the perception that professional fishing activities have been forced to fish closer to shore resulting in conflict between fishers and the general public. This negative impact was mostly perceived in PSGLMP.

## 3.8 Local economy domain

The economic domain is one of the more conventional domains for measuring human wellbeing, with GDP per capita growth at the national scale the most commonly used measure. The local economy domain includes the attributes: 1) employment and livelihoods 2) revenue in the local economy; and 3) intrinsic and bequest values. Three key positive impacts and one negative impact emerged from the analysis across both MPAs (Table 10).

**Table 10:** Local economy domain and associated attributes and corresponding social impacts (positive and negative) of Cape Byron and Port Stephens-Great Lakes Marine Parks.

Local economy domain			
Attribute	Impacts (positive or negative)	Number of coding references	Number of interviews coded at this theme (n = 58)
**Employment and livelihoods**	Increases employment opportunities	3	2
	Loss of business, e.g. professional fishers	29	16
**Revenue in the local economy**	Increases business opportunities or increase in revenue for existing businesses, e.g. increased tourist visitation	40	22
**Intrinsic and bequest values**	Intrinsic and bequest values enhanced by the marine park	30	21

The marine and coastal environment supports industries in CBMP and PSGLMP including fishing, aquaculture, marine tourism and recreation activities, and boating infrastructure. MPAs can provide economic opportunities to the local economy in particular for tourism, fishing and scientific research and management. In CBMP and PSGLMP, over one-third of the interviewees perceived that the tourism industry could play an essential role in contributing economically to the local area. As one participant commented:

So, by excluding fishing around Julian Rocks you’ve created a sanctuary, and then you notice a change. It creates breeding grounds that are undisturbed, it creates higher diversity because you are not taking particular stuff out and it creates higher density and that of course it makes more people want to go and have a look at it, so you create massive tourism value. (CBMP_1)

However, the parks can also be perceived as having a negative impact on the tourism sector, in particular for tourism relating to fishing. For example, one participant said:

It's a bream tournament, which is catch and release only. But the reason they don't come here anymore—and they've actually said that to us—is because it's too hard to work in regard to the marine park. And those particular tournaments do bring a lot to the local economy, that's for sure. (PSGLMP_24)

Local employment in natural resources relating to the MPAs and income generated is essential to understand whether members of the community can meet their basic needs. The MPA can generate employment for local communities, as one participant put it:

Obviously, that has a benefit to the business as well. People wouldn't come here to dive if the diving wasn't so good, or not as many people would come here to dive. So, from a business point of view, or as a professional, if it wasn't for the Marine Park maybe I wouldn't have the opportunity to be working here. (CBMP_8)

MPAs were perceived to negatively impact on some commercial fishers through the loss of access and impact on the wellbeing of fishers. As one participant said:

The marine park had one of the biggest impacts on us. Seal Rocks was hard enough, but when they put the marine park in at the sanctuary zone on Cape Hawke reef, that just devastated us. (PSGLMP_25)

The MPAs enhanced intrinsic and bequest values for about one-third of interviewees. Intrinsic and bequest values are considered as non-use economic value in this framework. Many participants stated that no-take zones protect marine biodiversity for future generations to enjoy, as well as to know that areas are being protected for their intrinsic value. For example, one interviewee said:

The marine park protects the environment; it helps keep it for our kids and our kid's children. And we know that stuffing up the planet is not leading to good things, we see it with climate change, and we've learnt that by maybe just going and raping and pillaging the planet that eventually it will bite us back. (CBMP_1)

## 4. Discussion

The development of this framework, involving thematic analysis to identify social impacts from the MPAs and interrelations between impacts, led to four main findings of social impacts in MPAs. First, local perspectives are crucial to understanding social impacts. So even while findings from other places and generic frameworks are useful, they cannot fully explain social impacts in particular places. Second, understanding social impacts gives insight into the nature of trade-offs that occur in decision-making regarding MPAs. Third, the intangible social impacts experienced by local communities are just as significant as the tangible ones for understanding how MPAs operate. Fourth, governance impacts have been the most influential factor affecting the social acceptability of the case study parks.

Prior studies have noted the importance of local perspectives and context when designing a wellbeing framework [54, 71] as wellbeing is multi-dimensional and intrinsically connected to the places and communities in which people live [72]. Furthermore, local perspectives should come from a wide range of interest/user groups, not just the extractive users, such as fishers, most obviously impacted [8]. Valuable insights into the social impacts of MPAs came from capturing impacts from a range of different groups about the co-occurrence of positive and negative social impacts. For example, opinions differed across interest/user groups on the impacts of the no-take zones.

There was a sense that the establishment of the parks increased the enjoyment of observing marine life at unfished sites, and also protecting biodiversity for future generations to enjoy. However, for extractive users’ loss of access is not just about the loss of physical access to fishing spots, but also has unintended consequences, including the loss of socialising with like-minded people. This unintended consequence is a significant impact on recreational fishers as spending time with family and friends is a crucial motivation to fish for recreational fishers in NSW [73]. The protection of biodiversity, at critical sites such as Julian Rocks, was a positive impact experienced by many of the participants. However, some participants also experienced negative impacts such as impacting of fisher’s safety by travelling further distances to fish and loss of access on the part of Aboriginal people to culturally significant places/marine resources. Our findings resonate with findings from other studies that the positive and negative impacts co-occur across the range of interest/user groups. It is essential to capture the complex web of co-existing positive and negative impacts to ensure impacts are equitable and shared amongst local communities [10].

Grasping the complex web of co-occurring positive and negative impacts across different user groups is the necessary groundwork to inform decisions about the trade-offs that have to be made in MPAs. The impacts around each domain of wellbeing are complex in that there are several attributes, with positive and negative impacts for each attribute. Outlining and categorising impacts allows different interest/user groups to understand the breadth of impacts. Evidence-based decision-making regarding trade-offs to meet the social objectives of the MPAs can then be made. A wellbeing framework allows for communities to understand the range of impacts experienced and that there are legitimate trade-offs that need to be made across a range of interest/user groups.

Social impact assessments have commonly focussed on tangible impacts, such as income. However, in this research, with the parks being in place for over a decade, economic benefits were not prioritised by participants, and seem not to be a key priority for local communities. A central finding from the research is that local communities’ experiences of the MPAs were most coloured by intangible impacts such as facilitating community cohesion, pride in the marine park or loss of cultural values and practices associated with the parks. These results are in accord with recent studies indicating the increasing acknowledgement and importance of intangible impacts in assessing social impacts of protected areas or environmental change, however, is an understudied area of research [e.g. 18, 74].

An important finding from this study is that the failure to address negative social impacts can undermine the legitimacy of MPAs. In particular, in the category of impacts we have called ‘governance’, participants felt there were significant deficits in terms of participatory decision-making, active and transparent monitoring and evaluation of management effectiveness to inform adaptive management. The governance domain had the most negative impacts [14] and only one positive impact coded to it. The number of interviews who raised governance issues was also consistently high, e.g. 40 (73%) of interviewees spoke about feeling ignored or disempowered by the implementation and ongoing management of the MPAs. The sentiment of feeling ignored and disempowered has undermined the legitimacy of the parks, exacerbated the negative impacts and inhibited the positive outcomes. Adaptive management and two-way communication between government and communities play a vital role in managing social impacts and addressing stakeholders’ concerns [17, 45]. This finding supports the work of other studies in the area linking the social impacts and how they are influencing the social acceptability of marine parks [e.g. 9].

Research linking wellbeing and the natural environment is still in its infancy even though wellbeing is progressively being adopted as an approach to policy development by governments, businesses and other organisations [e.g. 75, 76]. The contemporary concept of wellbeing is useful for providing linkages between human wellbeing and nature because it: 1) is a human-centred approach; 2) encompasses a broad range of indicators that can be used to measure community progress or impacts; and 3) captures subjective and relational as well as material aspects of wellbeing [25, 54, 72]. The conceptual framework helps unpack the complexities of wellbeing so that it can be included in a range of different policy settings. The framework can assist in the social process to work out trade-offs that may exist between different management options and may help people who have experienced negative impacts accept policy decisions. The wellbeing approach fits well with policy frameworks, is easily understandable by policymakers and stakeholders, and also by people working in disparate disciplines, such as economics, ecology and sociology.

### 4.1. Limitations

Further research is needed to ascertain whether the framework identified in this paper is appropriate for other MPAs. There is no way of knowing if CBMP and PSGLMP have similar social impacts to other MPAs in NSW, Australia or elsewhere. As the sample is small, we cannot claim the data presented here is representative of the broader population. A questionnaire survey could be used in further research to expand the sample size and to measure or rank perceptions of the impacts of MPAs. Nevertheless, combined with other studies, the findings of this research can contribute to a more comprehensive empirical understanding of the social impacts of MPAs. Further analysis is needed to understand factors beyond use types which are also important for understanding people’s perceptions of MPAs. Other factors, such as age, gender, are explored in the larger research project. Still, they are beyond the scope of this paper, which primarily lays out the domains of wellbeing from across the whole interviewee sample.

## 5. Conclusions

This research offers detailed insights into the social impacts of conservation interventions such as MPAs, with a framework of community wellbeing, which can be further explored with larger sample sizes and in other locations. A multi-dimensional community wellbeing framework is useful for capturing the diversity of social impacts perceived and experienced by coastal communities living adjacent to CBMP and PSGLMP. In our analysis, seven domains of wellbeing captured the breadth of impacts experienced by local communities: environment; health and safety; social connections; education and knowledge; culture and heritage; governance; and local economy.

It is well known that stakeholders have different scales of influence and power in decision-making [7]. Assessing what matters to local communities impacted by conservation interventions such as MPAs allows for a range of voices to be heard, across a diversity of interest/user groups. In this way, decision-makers have more context for understanding community experiences, rather than the loudest and most influential group unduly influencing how MPAs are perceived by the general public and by decision-makers. We provide a framework to be used by governments to work towards more effective, equitable and socially sustainable MPAs through much-needed monitoring of human dimensions of conservation interventions at the community level.

## References

[1] UNEP, editor The strategic plan for biodiversity 2011–2020 and the aichi biodiversity targets2010: Document UNEP/CBD/COP/DEC/X/2 Secretariat of the Convention on Biological Diversity, Nagoya, Japan.

[2] Spalding MD, Hale LZ. Marine protected areas: past, present and future—a global perspective In: Fitzsimons J, G W, editors. Big, Bold and Blue: Lessons from Australia’s Marine Parks Expansion,. Victoria, Australia: CSIRO; 2016.

[3] UNEP-WCMC and IUCN. Protected Planet Report 2016 UNEP-WCMC and IUCN: Cambridge UK and Gland, Switzerland; 2016.

[4] CBD. Quick guide to the Aichi Biodiversity Targets: protected areas increased and improved. In: Diversity CoB, editor. wwwcbdint/sp2013.

[5] Dudley N. Guidelines for applying protected area management categories: IUCN; 2008.

[6] Mascia MB, CLAUS C, Naidoo R. Impacts of marine protected areas on fishing communities. Conservation Biology. 2010;24(5):1424–9. 10.1111/j.1523-1739.2010.01523.x 20507354

[7] Sowman M, Sunde J. Social impacts of marine protected areas in South Africa on coastal fishing communities. Ocean & Coastal Management. 2018;157:168–79.

[8] Voyer M, Gladstone W, Goodall H. Understanding marine park opposition: the relationship between social impacts, environmental knowledge and motivation to fish. Aquat Conserv-Mar Freshw Ecosyst. 2014;24(4):441–62.

[9] Jones N, McGinlay J, Dimitrakopoulos PG. Improving social impact assessment of protected areas: A review of the literature and directions for future research. Environ Impact Assess Rev. 2017;64:1–7.

[10] Rasheed AR. Marine protected areas and human well-being–A systematic review and recommendations. Ecosystem Services. 2020;41:101048.

[11] Ban N, Gurney G, A. Marshall N, Whitney C, Mills M, Gelcich S, et al Well-being outcomes of marine protected areas. Nature Sustainability. 2019;2:524–32.

[12] Rogers DS, Duraiappah AK, Antons DC, Munoz P, Bai X, Fragkias M, et al A vision for human well-being: transition to social sustainability. Curr Opin Environ Sustain. 2012;4(1):61–73.

[13] Bennett NJ. Using perceptions as evidence to improve conservation and environmental management. Conservation Biology. 2016 10.1111/cobi.12681 26801337

[14] Franks P, Small R. Social Assessment for Protected Areas (SAPA) Methodology Manual for SAPA Facilitators London; 2016.

[15] Palmer Fry B, Agarwala M, Atkinson G, Clements T, Homewood K, Mourato S, et al Monitoring local well-being in environmental interventions: a consideration of practical trade-offs. Oryx. 2015;51(1):68–76.

[16] Oldekop J, Holmes G, Harris W, Evans K. A global assessment of the social and conservation outcomes of protected areas. Conservation Biology. 2015 10.1111/cobi.12568 26096222

[17] Fox HE, Mascia MB, Basurto X, Costa A, Glew L, Heinemann D, et al Reexamining the science of marine protected areas: linking knowledge to action. Conservation Letters. 2012;5(1):1–10.

[18] Schreckenberg K, Camargo I, Withnall K, Corrigan C, Franks P, Roe D, et al Social assessment of conservation initiatives. A review of rapid methodologies IIED. 2010.

[19] de Lange E, Woodhouse E, Milner-Gulland E. Approaches Used to Evaluate the Social Impacts of Protected Areas. Conservation Letters. 2016.

[20] Leisher C, Samberg L, Van Buekering P, Sanjayan M. Focal Areas for Measuring the Human Well-Being Impacts of a Conservation Initiative. 2013;5(3):997–1010. 10.1111/aogs.12042 23157497

[21] Fry BP, Agarwala M, Atkinson G, Clements T, Homewood K, Mourato S, et al Monitoring local well-being in environmental interventions: a consideration of practical trade-offs. Oryx. 2015:1–9.

[22] Schirmer J, Mylek M, Peel D, Yabsley B. People and Communities: The 2014 Regional Wellbeing Survey. University of Canberra: Canberra; 2015.

[23] Scrivens K, Smith C. Four Interpretations of Social Capital: An Agenda for Measurement. Paris: OECD Publishing; 2013.

[24] McGregor A. Wellbeing, Poverty and Conflict. ESRC Research Group on Wellbeing in Developing Countries, Briefing paper 1/08; 2008.

[25] Britton E, Coulthard S. Assessing the social wellbeing of Northern Ireland's fishing society using a three-dimensional approach. Marine Policy. 2013;37(0):28–36.

[26] Armitage D, Béné C, Charles AT, Johnson D, Allison EH. The Interplay of Well-being and Resilience in Applying a Social-Ecological Perspective. Ecology & Society. 2012;17(4):65–81.

[27] Gough I, McGregor JA. Wellbeing in developing countries: From theory to research: Cambridge University Press; 2007.

[28] King MF, Renó VF, Novo EM. The concept, dimensions and methods of assessment of human well-being within a socioecological context: a literature review. Soc Indic Res. 2014;116(3):681–98.

[29] Sen A. Development as Freedom: Oxford University Press; 1999.

[30] Sen A. The ends and means of sustainability. Journal of Human Development and Capabilities. 2013;14(1):6–20.

[31] Doyal L, Gough I. A theory of human need: Palgrave Macmillan; 1991.

[32] Gough I, McGregor IA, Camfield L. Theorizing wellbeing in international development In: McGregor IGaJA, editor. Wellbeing in Developing Countries: From Theory to Research: Cambridge University Press; 2007 p. 3–43.

[33] Costanza R, Fisher B, Ali S, Beer C, Bond L, Boumans R, et al Quality of life: An approach integrating opportunities, human needs, and subjective well-being. Ecol Econ. 2007;61(2–3):267–76.

[34] Gill DA, Cheng SH, Glew L, Aigner E, Bennett NJ, Mascia MB. Social Synergies, Tradeoffs, and Equity in Marine Conservation Impacts. Annual Review of Environment and Resources. 2019;44.

[35] Davies TE, Epstein G, Aguilera SE, Brooks CM, Cox M, Evans LS, et al Assessing trade-offs in large marine protected areas. PLoS one. 2018;13(4):e0195760 10.1371/journal.pone.0195760 29668750 PMC5905982

[36] Gollan N, Voyer M, Jordan A, Barclay K. Maximising community wellbeing: Assessing the threats to the benefits communities derive from the marine estate. Ocean & Coastal Management. 2019;168:12–21.

[37] Biedenweg K, Stiles K, Wellman K. A holistic framework for identifying human wellbeing indicators for marine policy. Marine Policy. 2016;64:31–7.

[38] Kaplan-Hallam M, Bennett NJ. Adaptive social impact management for conservation and environmental management. Conservation Biology. 2017;00(0,1–11):n/a–n/a. 10.1111/cobi.12985 29063710

[39] Chaigneau T, Brown K, Coulthard S, Daw TM, Szaboova L. Money, use and experience: Identifying the mechanisms through which ecosystem services contribute to wellbeing in coastal Kenya and Mozambique. Ecosystem Services. 2019;38:100957.

[40] Camfield L. The why and how of understanding 'subjective well-being: Exploratory work by the WeD Group in four developing countries. WeD Working Paper No 26 University of Bath, Bath. 2006.

[41] Camfield L, Crivello G, Woodhead M. Wellbeing Research in Developing Countries: Reviewing the Role of Qualitative Methods. Soc Indic Res. 2009;90(1):5–31.

[42] Newig J, Kvarda E. Participation in environmental governance: legitimate and effective? Environmental Governance: Edward Elgar Publishing; 2012.

[43] Reed MS, Graves A, Dandy N, Posthumus H, Hubacek K, Morris J, et al Who's in and why? A typology of stakeholder analysis methods for natural resource management. Journal of Environmental Management. 2009;90(5):1933–49. 10.1016/j.jenvman.2009.01.001 19231064

[44] Brooks K, Barclay K, Grafton RQ, Gollan N. Transforming coastal and marine management: Deliberative democracy and integrated management in New South Wales, Australia. Marine Policy. 2020:104053.

[45] Vanclay F, A E, I A, D F. Social Impact Assessment: Guidance for assessing and managing the social impacts of projects International Association for Impact Assessment; 2015.

[46] Yin R. Case study research: Design and methods 4th ed2009.

[47] Creswell JW. Qualitative inquiry and research design: Choosing among five approaches: Sage; 2013.

[48] Miles MB, Huberman AM. Qualitative data analysis: An expanded sourcebook: Sage; 1994.

[49] Blaikie N. Designing social research: Polity; 2009.

[50] Braun V, Clarke V. Using thematic analysis in psychology. Qualitative Research in Psychology. 2006;3(2):77–101.

[51] Voyer M, Gollan N, Barclay K, Gladstone W. ‘It׳s part of me’; understanding the values, images and principles of coastal users and their influence on the social acceptability of MPAs. Marine Policy. 2015;52(0):93–102.

[52] Stiglitz J, Sen A, Fitoussi JP. Report by the Commission on the Measurement of Economic Performance and Social Progress. 2009.

[53] Breslow SJ, Sojka B, Barnea R, Basurto X, Carothers C, Charnley S, et al Conceptualizing and operationalizing human wellbeing for ecosystem assessment and management. Environmental Science & Policy. 2016.

[54] McGregor A, Coulthard S, Camfield L. Measuring what matters: the role of well-being methods in development policy and practice. 2015.

[55] Kingsley J, Townsend M, Henderson-Wilson C, Bolam B. Developing an Exploratory Framework Linking Australian Aboriginal Peoples’ Connection to Country and Concepts of Wellbeing. International Journal of Environmental Research and Public Health. 2013;10(2):678 10.3390/ijerph10020678 23435590 PMC3635170

[56] Feary S. Sea Countries of New South Wales: a benefits and threats analysis of Aboriginal people's connections with the marine estate. 2015.

[57] Young, Foale S, Bellwood D. Why do fishers fish? A cross-cultural examination of the motivations for fishing. Marine Policy. 2016;66:114–23.

[58] Abraham A, Sommerhalder K, Abel T. Landscape and well-being: a scoping study on the health-promoting impact of outdoor environments. International Journal of Public Health. 2010;55(1):59–69. 10.1007/s00038-009-0069-z 19768384

[59] Research Sweeney. Marine Estate Community Survey Final Report. 2014.

[60] Alder J. Costs and effectiveness of education and enforcement, Cairns Section of the Great Barrier Reef Marine Park. Environ Manage. 1996;20(4):541–51. 10.1007/BF01474654 8661613

[61] Angulo-Valdes JA, Hatcher BG. A new typology of benefits derived from marine protected areas. Marine Policy. 2010;34(3):635–44.

[62] Bennett NJ, Di Franco A, Calò A, Nethery E, Niccolini F, Milazzo M, et al Local support for conservation is associated with perceptions of good governance, social impacts, and ecological effectiveness. Conservation Letters. 2019;0(0):e12640.

[63] AIMS. The AIMSindex of marine industry Australian Institute of Marine Science; 2016.

[64] Kelaher BP, Page A, Dasey M, Maguire D, Read A, Jordan A, et al Strengthened enforcement enhances marine sanctuary performance. Global Ecology and Conservation. 2015;3:503–10.

[65] Harasti D, Malcolm H, Gallen C, Coleman MA, Jordan A, Knott NA. Appropriate set times to represent patterns of rocky reef fishes using baited video. Journal of Experimental Marine Biology and Ecology. 2015;463:173–80.

[66] Harasti D, Martin-Smith K, Gladstone W. Does a No-Take Marine Protected Area Benefit Seahorses? PLoS ONE. 2014;9(8):e105462 10.1371/journal.pone.0105462 25137253 PMC4138119

[67] Hammerton Z, Bucher D, Page A. The influence of protection and temperature on subtropical reef fish assemblages in Cape Byron Marine Park Aquatic Conservation: Marine and Freshwater Ecosystems 2019.

[68] Marine Parks Authority. Cape Byron Marine Park Operational Plan In: Authority NMP, editor. Sydney: Department of Environment, CLimate Change and Water; 2010.

[69] Marine Parks Authority. Port Stephens-Great Lakes Marine Park Operational Plan In: Authority NMP, editor. Sydney 2010.

[70] Government NSW. Government response to the Report of the Independent Scientific Audit of Marine Parks in New South Wales—A new approach to managing the NSW marine estate NSW Department of Primary Industries; 2013.

[71] Narayan D, Chambers R, Kaul Shah M, Petesch P. Voices of the poor-Crying out fo change New Yourk: Oxford: University Press for the World Bank; 2000.

[72] White SC. Introduction: The Many Faces of Wellbeing In: White SC, Blackmore C, editors. Cultures of Wellbeing: Method, Place, Policy. London: Palgrave Macmillan UK; 2016 p. 1–44.

[73] West L, Stark K, Murphy J, Lyle J, Ochwada-Doyle F. Survey of Recreational Fishing in New South Wales and the ACT, 2013/14. 2015.

[74] Breslow SJ, Allen M, Holstein D, Sojka B, Barnea R, Basurto X, et al Evaluating indicators of human well-being for ecosystem-based management. Ecosystem Health and Sustainability. 2017:1–18.

[75] ABS. Measures of Australia's Progress 2013. 2013.

[76] McLeod K. Our people-multidimensional wellbeing in New Zealand. Analytical Paper; 2018. Report No.: 1988556856.

